# Supplementary material for: Temporal dynamics of proteome and phosphorproteome during neuronal differentiation in the reference KOLF2.1J iPSC line
Source: bioRxiv. 2025 Mar 26:2025.03.25.645331. Preprint. [Version 1] doi: 10.1101/2025.03.25.645331 (PMC12190317; doi:10.1101/2025.03.25.645331)

bioRxiv preprint doi: https://doi.org/10.1101/2025.03.25.645331; this version posted March 26, 2025. The copyright holder for this preprint (which was not certified by peer review) is the author/funder. This article is a US Government work. It is not subject to copyright under 17 USC 105 and is also made available for use under a CC0 license.

Supplementary materials

Figure S1: Optimization of phosphopeptide enrichment analysis

(A) Comparison of phosphopeptide enrichment efficiency across different commercial beads. Welch's t test with $*P < 0.05$.

(B) Effect of input protein amount on phosphopeptide identification. Welch's t test with $*P < 0.05$.

(C) Coefficient of variation (CV) analysis for phosphopeptide quantification across different input protein amounts.

(D) Missing values of protein identification across the differentiation.

(E) Missing values of phosphopeptide identification across the differentiation.

(F) Venn diagram illustrating the overlap of the proteome and phosphoproteome overall identified in KOLF2.1J-derived neurons.

(G) Distribution of phosphorylated residues.

Figure S2: Characterization of neuronal markers

(A) Immunofluorescence staining of pluripotency and neuronal markers at different stages of differentiation. Scale bars represent 25 μm.

(B) Protein quantification of different types of neuronal markers during differentiation. Log_2 fold change (DN/D0) was calculated relative to Day 0 (iPSC) with error bars representing mean ± SD.

Figure S3: Dynamic trends in total protein and phosphosite levels within functional pathways

(A) Heatmap showing the dynamics of microtubule-associated proteins and their phosphosites.

(B) Spearman correlation analysis between phosphosites and their corresponding unmodified proteins.

(C) Discordant trends between the two omics data involve the RNA transport and localization pathway.

Figure S4: Time-point comparisons of kinase activity based on KSEA

(A) Bar plot showing the enrichment scores based on KSEA analysis comparing day D4 vs D0

(A) Bar plot showing the enrichment scores based on KSEA analysis comparing day D7 vs D0

(A) Bar plot showing the enrichment scores based on KSEA analysis comparing day D14 vs D0

(A) Bar plot showing the enrichment scores based on KSEA analysis comparing day D21 vs D0

(A) Bar plot showing the enrichment scores based on KSEA analysis comparing day D28 vs D0

Positive enrichment scores (orange bars) indicate kinases with increased activity, negative enrichment scores (blue bars) indicate kinases with decreased activity.

bioRxiv preprint doi: https://doi.org/10.1101/2025.03.25.645331; this version posted March 26, 2025. The copyright holder for this preprint (which was not certified by peer review) is the author/funder. This article is a US Government work. It is not subject to copyright under 17 USC 105 and is also made available for use under a CC0 license.

Table S1 Protein abundance in the proteomics data.

Table S2 Phosphopeptide abundance in the phosphoproteomics data.

Table S3 Kinase substrate enrichment score files

bioRxiv preprint doi: https://doi.org/10.1101/2025.03.25.645331; this version posted March 26, 2025. The copyright holder for this preprint (which was not certified by peer review) is the author/funder. This article is a US Government work. It is not subject to copyright under 17 USC 105 and is also made available for use under a CC0 license.

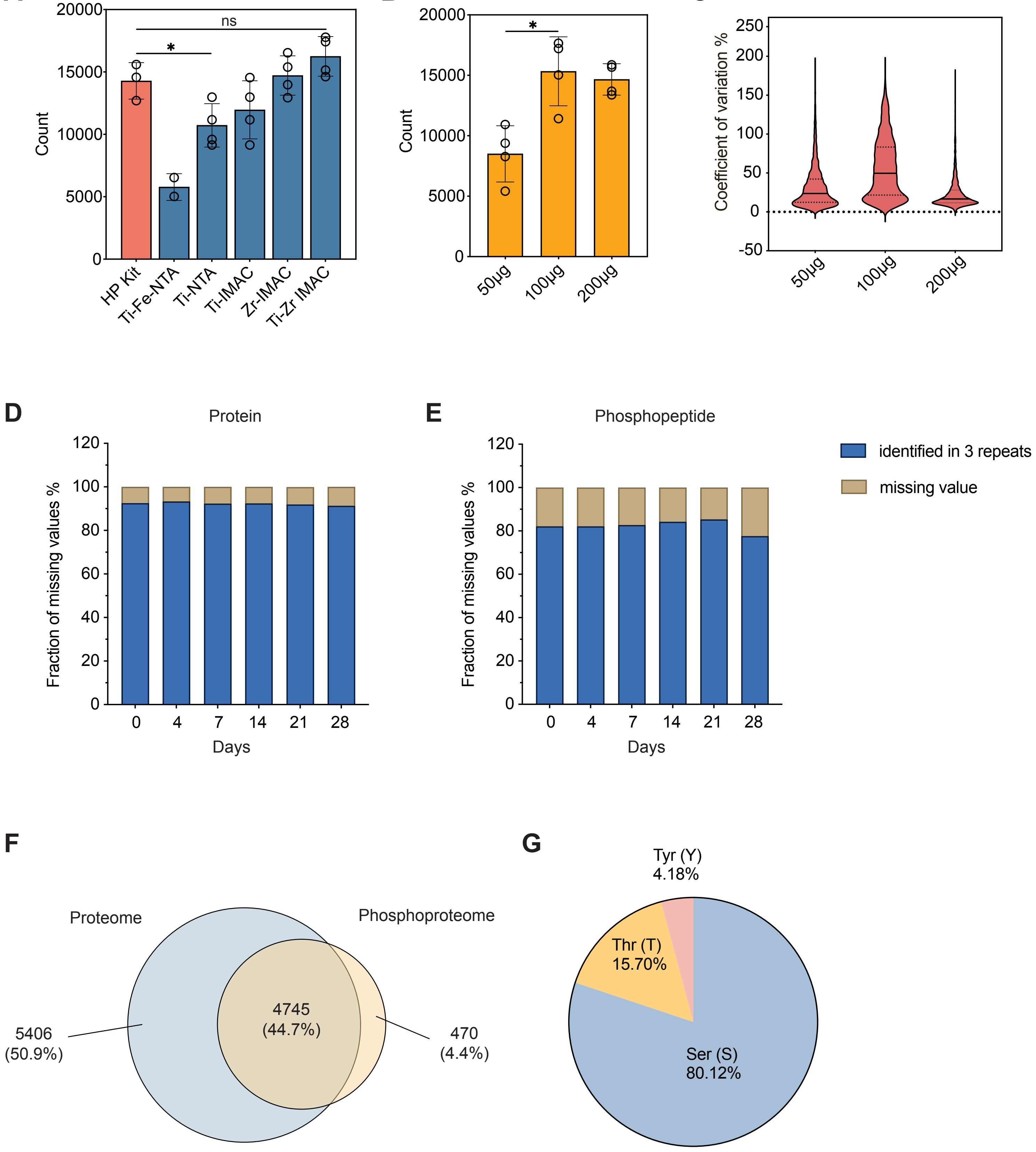

bioRxiv preprint doi: https://doi.org/10.1101/2025.03.25.645331; this version posted March 26, 2025. The copyright holder for this preprint (which was not certified by peer review) is the author/funder. This article is a US Government work. It is not subject to copyright under 17 USC 105 and is also made available for use under a CC0 license.

A

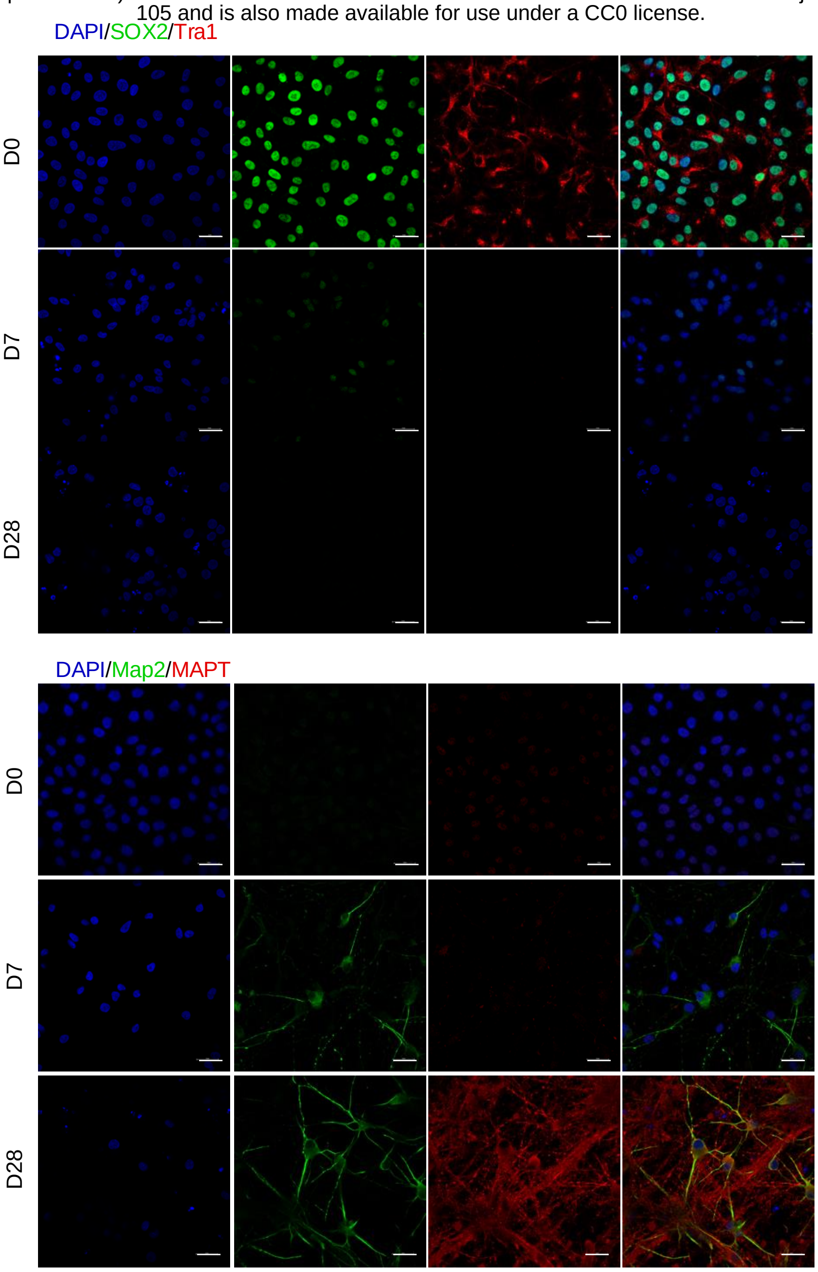

B

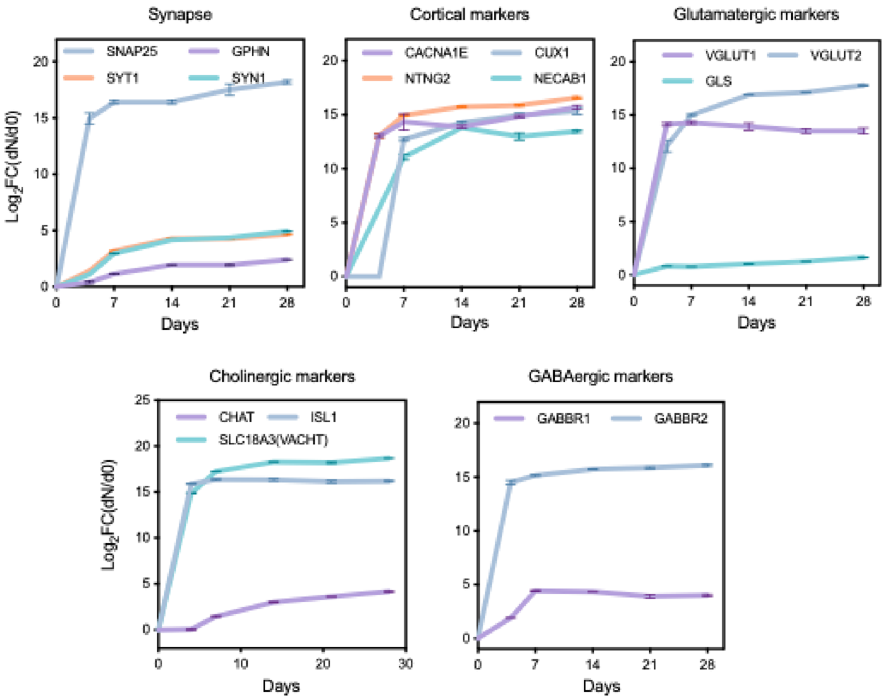

bioRxiv preprint doi: https://doi.org/10.1101/2025.03.25.645331; this version posted March 26, 2025. The copyright holder for this preprint (which was not certified by peer review) is the author/funder. This article is a US Government work. It is not subject to copyright under 17 USC 105 and is also made available for use under a CC0 license.

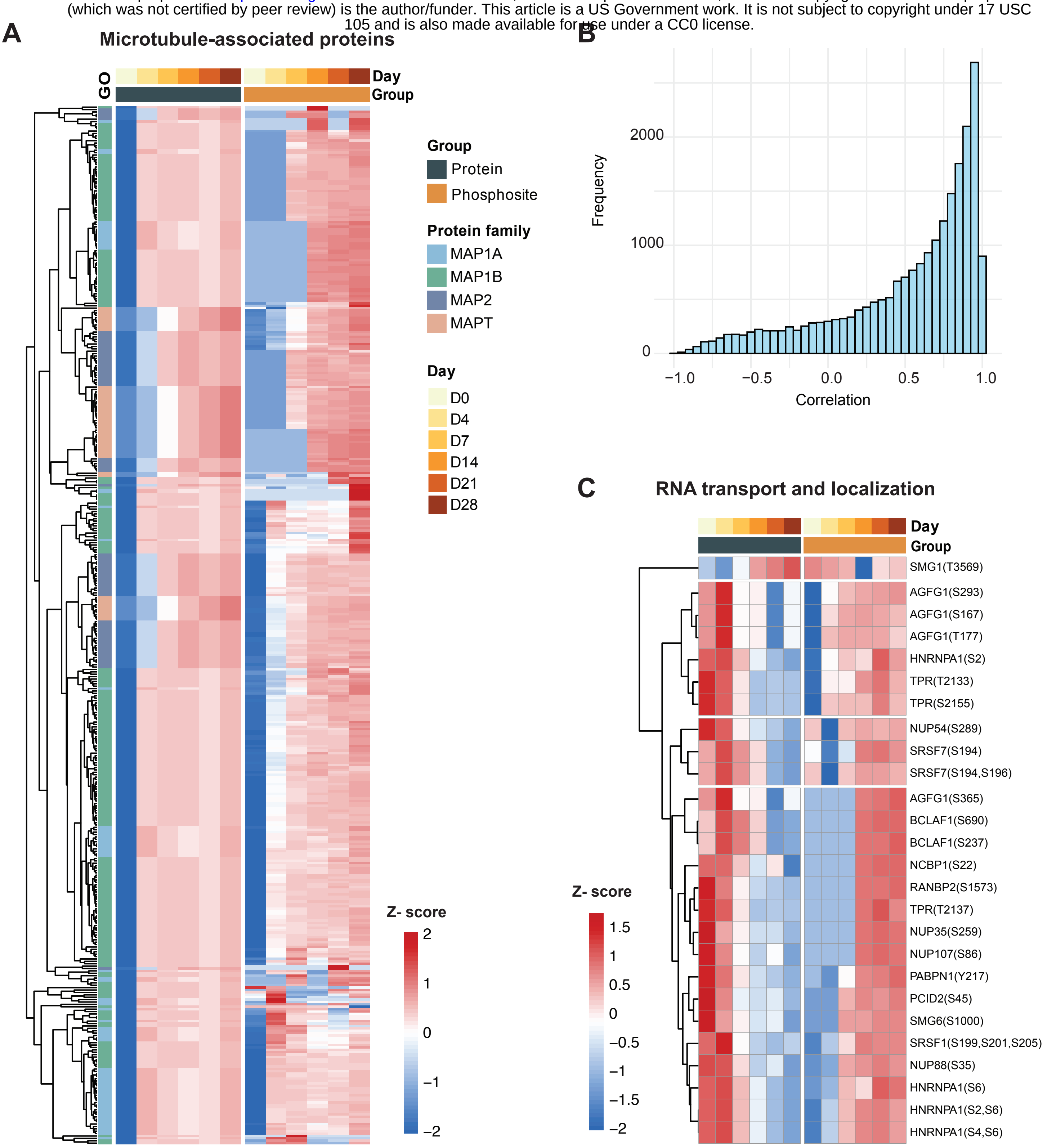

bioRxiv preprint doi: https://doi.org/10.1101/2025.03.25.645331; this version posted March 26, 2025. The copyright holder for this preprint (which was not certified by peer review) is the author/funder. This article is a US Government work. It is not subject to copyright under 17 USC 105 and is also made available for use under a CC0 license.

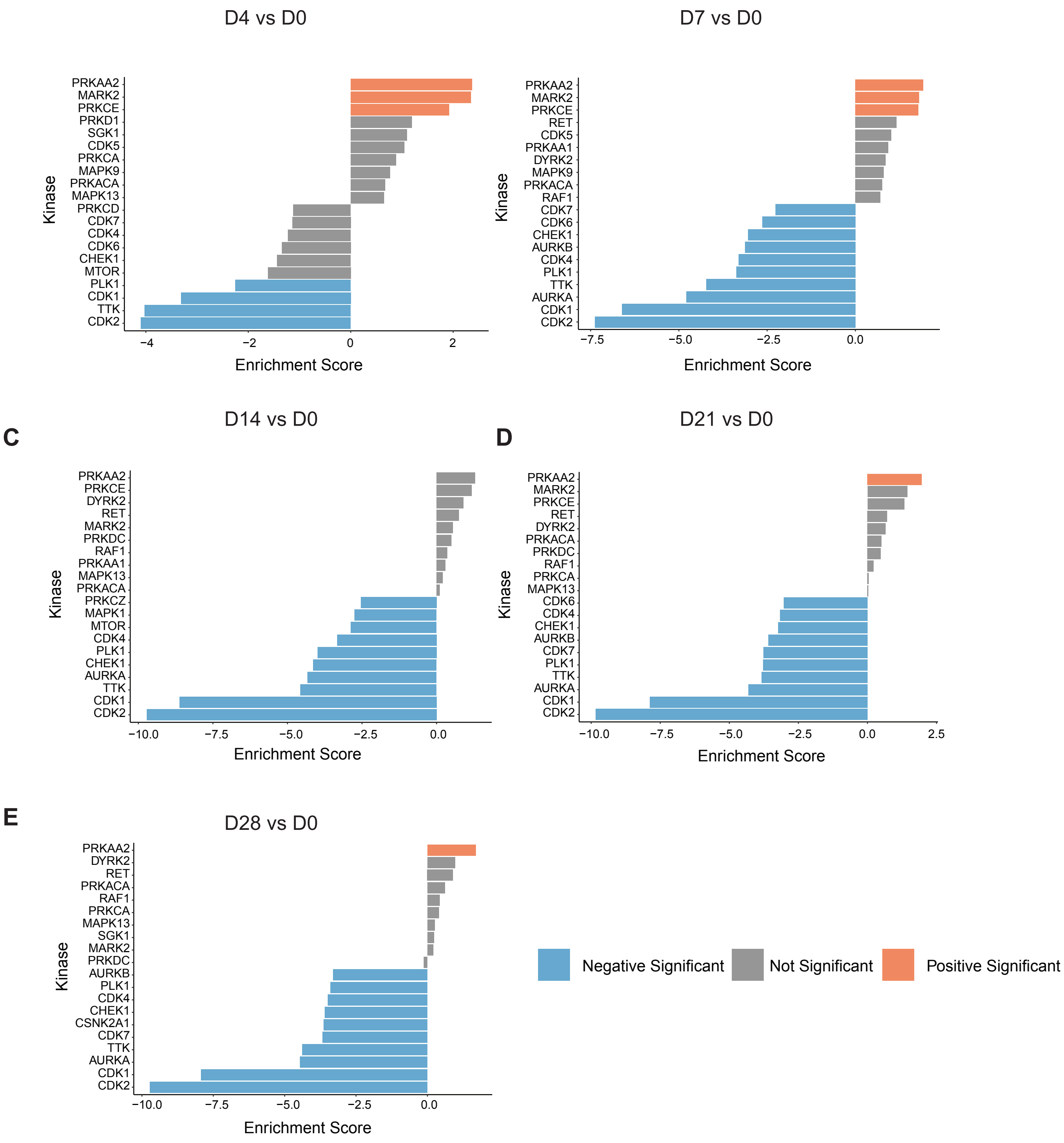

Supplement: Supplement 1 [file NIHPP2025.03.25.645331v1-supplement-1.pdf]
